# Application of a Vision-Based Single Target on Robot Positioning System

**DOI:** 10.3390/s21051829

**Published:** 2021-03-05

**Authors:** Jing Yu, Wensong Jiang, Zai Luo, Li Yang

**Affiliations:** School of Metrology and Test Engineering, China Jiliang University, Hangzhou 310018, China; p1802085272@cjlu.edu.cn (J.Y.); luozai@cjlu.edu.cn (Z.L.); lyang@cjlu.edu.cn (L.Y.)

**Keywords:** monocular vision, target recognition, cross-ratio invariance, single-target mobile robot positioning

## Abstract

In this paper, we propose a Circular-ring visual location marker based on a global image-matching model to improve the positioning ability in the fiducial marker system of a single-target mobile robot. The unique coding information is designed according to the cross-ratio invariance of the projective theorem. To verify the accuracy of full 6D pose estimation using the Circular-ring marker, a 6 degree of freedom (DoF) robotic arm platform is used to design a visual location experiment. The experimental result shows in terms of small resolution images, different size markers, and long-distance tests that our proposed robot positioning method significantly outperforms AprilTag, ArUco, and Checkerboard. Furthermore, through a repeatable robot positioning experiment, the results indicated that the proposed Circular-ring marker is twice as accurate as the fiducial marker at 2–4 m. In terms of recognition speed, the Circular-ring marker processes a frame within 0.077 s. When the Circular-ring marker is used for robot positioning at 2–4 m, the maximum average translation error of the Circular-ring marker is 2.19, 3.04, and 9.44 mm. The maximum average rotation error is also 1.703°, 1.468°, and 0.782°.

## 1. Introduction

Mobile robots with visual positioning technology have the ability of environmental cognition. Such robots can complete a task upon providing the required information, such as augmented reality [1] and robot navigation [2,3]. For a single-target mobile robot, the available information determines its mode of motion. To ensure the normal operation of a robot, the pose estimation must be accurate and provided in real time [4].

The central problem of mobile robots is an accurate and reliable position estimation. External positioning systems are widely used in the mobile robot field. The system can measure the real position of mobile robots. The external positioning systems include types such as radio or ultrasound. The system is expensive to maintain and too bulky for small robots. Artificially designed positioning systems such as the circular tag [5,6] and square tag [7] provide valid and accurate feature points for pose estimation. Two special diameters are proposed in [8] for estimating the normal vector and the position of a circle, which can be used to identify the pose of the target. The square tag serves as a fiducial marker that accurately obtains the pose information. This method is designed so that the target’s four corners can be easily detected. A checkerboard can be used to provide target pose information. In [9,10], a method with pairwise parallel and intersecting relationships of a four-point coplanar was used to solve the positioning problem.

The design of the positioning systems faces a problem. They need to choose one or more different priorities on detection distance, positioning accuracy, recognition rate, and recognition speed. Therefore, the above-mentioned targets are usually not widely used in various scenarios. Until the advent of the fiducial marking system, the positioning of the robot can be achieved by identifying the pose information of the marker. The fiducial marking system was successfully applied in this field. In order to improve the detection distance and accuracy, the use of circular forms as fiducial marks has gradually become the mainstream. In general, these circular reference markers lack the ability to estimate full 6D pose information. They do not provide the user with the position and orientation of the marker relative to the camera. In this paper, the concentric ring is used as the component of the visual marker, and the center of the ring is used as the feature point to solve the pose information. The marker has the advantages of high measurement accuracy at a long distance, high calculation efficiency, and strong robustness against strong light.

This work presents contributions to robot vision positioning. The main contribution lies in the design of a new Circular-ring cooperation target and the marker identification process. The Circular-ring marker can be easily printed on paper and meets the general design principles of visual reference marking, it takes the center of the Circular ring as the marker point due to the less expensive centroid operation. The square markers usually have a binary code, and the Circular-ring marker sets the feature points cross-ratio value as the unique coding to ensure rotation invariance [11]. The Canny edge detection [12] and the Floodfill algorithm [13] are used in the recognition process of the Circular-ring marker; they provide continuous edges for ellipse fitting. In global image detection, the identification process takes a short time. The experimental test of the Circular-ring marker takes 77 ms/frame. It can be used in real-time occasions. Moreover, this article takes the average of the two ellipse centers as the observation point, which can eliminate the center offset error [14]. In our proposed setting, an industrial camera takes several images and performs global matching to identify the center of the Circular ring. The performance of the Circular-ring marker is demonstrated by the comparison experiments with the AprilTag2, ArUco, and checkerboard marker systems.

The present work is structured as follows. In Section 2, an overview of the related work on various visual markers has been discussed. The section describes the design concept, identification method, and application range of different markers. Section 3 describes the general principles of visual fiducial marker design. The section designs a reasonable Circular-ring marker based on these general principles; then, it introduces details regarding the concept of cross-ratio for the Circular-ring marker. Section 4 describes the image preprocessing process of the Circular-ring marker and then uses the Union-Find algorithm [15] to obtain continuous edges. Then, the continuous edges will be used to fit the ellipse. Finally, the section explains the decoding process of the Circular-ring marker. In Section 5, each parameter of the Circular-ring marker is determined by experiments. Then, the performance of AprilTag, ArUco, Checkerboard, and the Circular-ring marker are compared through different experiments. Section 6 summarizes the whole article and discusses the shortcomings and prospects of this article.

## 2. Related Works

To meet the requirement for a single-target robot based on vision markers, as discussed above, several markers have been developed for positioning. The idea was to focus on the development of passive visual markers because they are cheap to make and easy to use. The QR Code is one of the most well-known passive markers [16]. The marker can store a lot of information, so it is suitable for transferring data. In addition, the barcode is partially damaged, but it can correctly decode the information according to the in-built error correction code. Another example of a QR code is the augmented reality marker, which has the advantage of being more easily used for target tracking. Among these markers, ARTag [17] and Artoolkit [18] are used more frequently. The detection method of the ARToolkit marker is fixed threshold binarization. This method is fast, but it does not adapt to the light changes. The ARTag marker improves the robustness, and its detection method is based on the image gradient. Another marker based on the same approach as ARToolKit and ARTag is ArUco. The ArUco has a small false-positive rate in case of the occlusion problem. It generates encoding information according to criteria that maximize the marker distance and the number of bit conversions. Garrido et al. proposed an ArUco for augmented reality [19]. Kam et al. used a linear Kalman filter to improve the robustness of the ArUco [20]. This ArUco marker has good real time and robustness, which makes it an ideal choice to compare with our marker. The University of Michigan’s April laboratory also designed an efficient and robust AprilTag. The AprilTag utilizes a square marker design with a 2D barcode, which is recognizable from short and long distances. However, this method has a lower recognition speed. To solve this problem, Olson et al. proposed a second version, AprilTag2 [21]. The AprilTag2 improved performance on small images allows the use of decimated input images, resulting in gains in detection speed. The AprilTag2 marker will also be compared.

The square markers are successful in the field of visual tracking. However, the circular feature is gradually used to measure the target pose. For example, the circular ring on an artificial landmark enables recognition and accurate position. Zhang et al. used circular spots to form markers [22] and used inter-frame data for relative pose estimation. However, his experiments showed that the errors of the smoothed pose estimates are below 5% (position) and 10° (orientation). In [23], a concentric circle marker with alternating black and white circles is proposed, which achieves the purpose of detection by identifying the connection between the centroids of the ellipse. In the same way, it is shown in the literature [24] that the marker is composed of multiple circles. The CircularTag marker uses a circular nature and non-linear optimization to estimate the marker’s pose. The CircularTag has high positioning accuracy, but its shape is complicated. The WhyCon [25] marker includes two circles to simplify the shape. Although the marker sacrifices positioning accuracy, the recognition distance is increased. The literature [14] indicates that the center positioning accuracy is better when the inner and outer radius is 1:2, and this suggestion is also taken in the marker of this article.

The markers lack an estimate of the full 6D pose, as discussed above. By exploiting the projective properties of a circle set of sizeable dots, Bergamasco et al. proposed a high-precision RUNE-Tag marker [26]. The design method of the marker focuses on allowing greater occlusion robustness. Therefore, the identification experiment can only be completed at close range. The TRIP marker [27] consists of several concentric circles. The concentric circles are divided into several angular regions and painted black or white. The marker can achieve an accuracy of 1 to 3% relative error, but the disadvantage is the high computational cost. Patruno et al. [28] proposed a solution for the landing of Unmanned Aerial Vehicle (UAV) on an artificial marker. The marker can estimate pose by an H-shape in the circular ring. The results show that the marker is 1 m away from the UAV, and the average Root Mean Squared Error values of 13.7 mm and 1.04° in the position and orientation estimations have been found, respectively. The marker detection rate can be improved by setting different coding modes. For example, a Visual–Inertial Self-Tracker marker [29] can design thousands of different code systems by adding a dedicated “data ring” in the circle. The system is mainly aimed at large-scale tracking and can be identified by a wide-angle camera. Mooser’s TriCodes marker [30] is designed for large, dynamic sets of fiducials and provides vast pattern libraries. They prove the TriCodes marker’s superiorities in the presence of large numbers of feature points. However, the Whycode marker is used for a small number of feature points. The WhyCode [31] uses an open-ended “necklace encoding” based on the WhyCon, so it can perform a full 6D pose estimation. The accuracy of the WhyCode marker is at the centimeter level, and its coverage area is large in cases concerning the location of multi-robots. However, using the WhyCode marker results in a long processing time for the first frame. The efficiency is reduced when the marker reappears after suddenly disappearing. The paper [32] recommends the use of a cross-ratio between detection points, including our marker in this article. The advantage lies in the use of projection invariance, which can be recognized without any correction of the image. Our marker is designed with multiple circular rings to achieve the purpose of the full 6D pose estimation. It is a disadvantage that the marker occupies a large area, but the improvement of the pose estimation accuracy may counteract this disadvantage.

## 3. Designing the Encoded Marker Points

**Figure 1:**
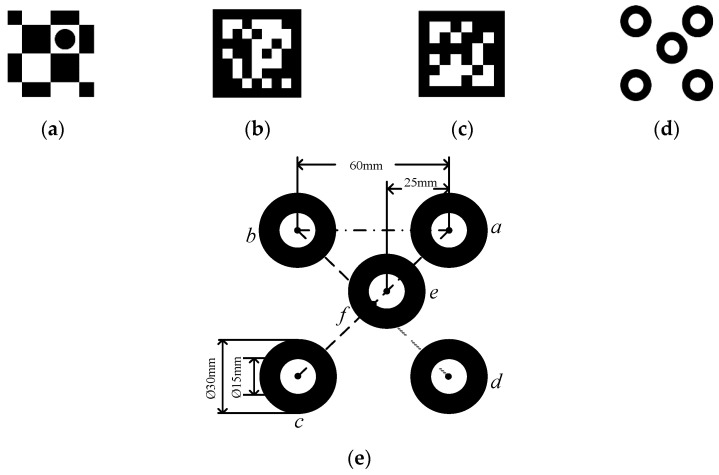
Four markers: (**a**) Checkerboard; (**b**) ArUco; (**c**) AprilTag; (**d**) Circular ring; (**e**) The proposed Circular-ring marker size design.

Visual fiducial location systems are widely used in the field of robotics. The visual fiducial markers help extract accurate feature points from the camera projection images for pose estimation. These targets are shown in Figure 1. A visual fiducial marker must be recognized quickly and accurately. This paper designs an encoding marker point based on the following design principles:

(1) The markers must be correctly detected in the case of long-distance work, illumination variation, and complex background.
(2) The markers must be able to encode specific information to be used after being captured by the camera.
(3) The markers should be easy to use and cost-effective. For instance, a marker should be able to be printable on paper and recognized using ordinary grayscale cameras.
(4) The fundamental function of a marker is to provide an accurate position of the object, where a 6 DoF includes both translation and rotation.
(5) When a scanning probe is operating at the end of a robotic arm, the scanning probe’s information and the location of its end-effector must be synchronized. To facilitate this the process of reading, the markers should be fast enough to meet this synchronicity.

Based on the above design principles, here, we use the advantages of a circular-ring including easiness of being separated from the captured image, fast recognizability, and stability of its center point identification. The Circular-ring marker shown in Figure 1d is designed. These five concentric circles of the same size make up the Circular-ring marker. We further set those center points as the encode marker points, a,b,c,d,e, where a,c,e are collinear marker points. Figure 1e illustrates the size and relative position of the circular rings.

**Figure 2:**
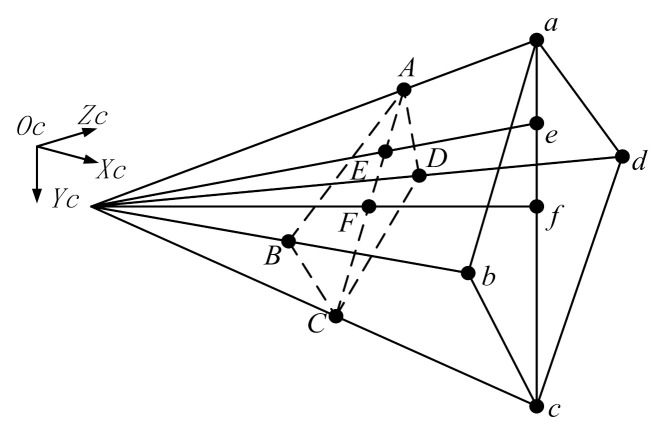
Cross-ratio invariance.

This paper designs a unique encoding for the Circular-ring marker. The cross-ratio invariance of the projective theorem is used to determine the location of these encoded marker points. In Figure 2, the point f in the camera projection diagram is the intersection of the two-line segments. The cross-ratio of those points a,e,f,c is

(1) $$R(a,e,f,c)=\frac{\left|\vec{af}\right|\times \left|\vec{ec}\right|}{\left|\vec{ef}\right|\times \left|\vec{ac}\right|}.$$

Here, we obtain A,E,F,C by the camera projection image; hence, the cross-ratio of those points remains unchanged and holds

(2) $$R(A,E,F,C)=\frac{\left|\vec{AF}\right|\times \left|\vec{EC}\right|}{\left|\vec{EF}\right|\times \left|\vec{AC}\right|}=R(a,e,f,c).$$

The cross-ratio invariance ensures accurate identification of the Circular-ring marker.

## 4. Identifying the Circular-Ring Marker

To realize the Circular-ring marker detection, a low computational cost detection method should be applied, such as the frame difference accumulation method in [33], the parallel pixel scanning method in [34,35], and the Union-Find algorithm in [15]. Those three methods are compared according to the detailed situation in our reach. In detail, the rotor visualization can be achieved by frame difference accumulation. It uses the Hough transform to detect the circle and then applies the adjusted value to quickly obtain the ellipse. The Circular-ring marker includes two concentric circles. Between adjacent frames, the pixels inside the circular ring have little change. The use of the frame difference accumulation method will cause Canny edge detection to produce double edges. When we use the Hough transform for circle detection, it is difficult to select the minimum distance between the centers of the detected circles. If the parameter is too small, multiple neighbor circles may be falsely detected in addition to a true one. If it is too large, some circles may be missed. [34] uses parallel pixel scanning to accelerate ellipse detection, and they greatly reduce the computation time. They use the Local Projection Profile method to correct the tilt of the text in the preprocessing process. The literature [35] mentions that the LPP algorithm has high accuracy, but the detection range is from −10° to 10°. However, the tilt of the Circular-ring marker more than 10° occurs frequently in practice. The Union-Find algorithm can quickly obtain all continuous edges; it is not subject to the above conditions. For this reason, the Union-Find algorithm is selected for edge segmentation in our research.

### 4.1. Image Preprocessing

**Figure 3:**
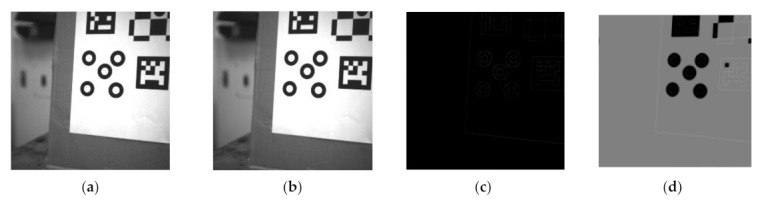
(**a**) The input image; (**b**) Gaussian blurred image; (**c**) Canny edge detection image; (**d**) Floodfill image.

In the captured image, the edge information extraction method is used to determine the recognition rate of the encoded marker. The input image is a grayscale image as shown in Figure 3a. In some image edge detection methods, the common edge detection templates include the Sobel operator and the log operator. However, the techniques are sensitive to noise and poor continuity within the captured image. The Canny operator addresses these issues [12]. In the Canny algorithm, a Gaussian smoothing filter is used to filter the image; see Figure 3b. The non-maximum suppression and double threshold mechanism can be used to obtain edge information in the image, as shown in Figure 3c.

The flooding algorithm [13] is used to fill the connected domains of the image. The (0,0) coordinate point on the image is set as the initial point and then spread from the initial point to the nearby pixels; then, it is assigned the pixel value to 127 to produce Figure 3d. Finally, the area with a pixel value of 127 is deleted and excluded them from subsequent detection.

### 4.2. Image Edge Segmentation

The Floodfill image is used for edge segmentation. To obtain continuous contour points, edge tracking is often used to analyze the topological structure of the binary images. However, for a given edge, this method may result in detecting multiple edges.

The Union-Find algorithm is used for edge segmentation. The algorithm traverses the black and white pixels in the Floodfill image. Then, we can obtain all the edges by the eight-connected search method.

We further assign different characteristic values to each edge, i.e., the pixel value of the coordinate point (m,n) is f(m,n) in the Floodfill image. Then, this method traverses all edges to the coordinate point (x,y). Then, the coordinate point (x+i,y+j) is obtained following the step illustrated in the eight-connected search method. Then, the coordinate point (X,Y) is recorded if f(x+i,y+j)+f(x,y)=255. The coordinates hold the following

(3) $$\left\{\begin{array}{l} X=2x+i\;Y=2y+j\\ g_{x}=i\times (\;f(x+i,y+j)-f(x,y))\\ g_{y}=j\times (\;f(x+i,y+j)-f(x,y)) \end{array}\right.$$

where $g_{x}$ and $g_{y}$ are set to zero in cases where they are obtained as negative numbers. $g_{x}$ and $g_{y}$ are intermediate quantities; they are used to calculate the characteristic value G. Note that in our formulation, (i,j) adopts one of the tuples in $\left\{(-1,1);(0,1);(1,1);(1,0)\right\}$. The next step is to obtain the maximum and minimum values of the coordinates on the same edge, which are $X_{\max},X_{\min},Y_{\max},Y_{\min}$. According to Formula (3), the *k*-th coordinate point $\left(X_{k},Y_{k}\right)$ contains information $g_{x}{}_{k}$ and $g_{y}{}_{k}$, which satisfies

(4) $$\left\{\begin{array}{l} \bar{X}=(X_{\max}+X_{\min})/2+\Delta x\\ \bar{Y}=(Y_{\max}+Y_{\min})/2+\Delta y\\ G=\sum_{k=1}^{n}\left((X_{k}-\bar{X})\times g_{x}{}_{k}+(Y_{k}-\bar{Y})\times g_{y}{}_{k}\right) \end{array}\right.$$

where Δx=0.0511 and Δy=−0.0285 are used to adjust the center pixel point $\left(\bar{X},\bar{Y}\right)$. Add noise to the center coordinates. Δx and Δy can be arbitrarily set to a smaller number whose absolute value is less than 1. At the same edge, n is the total number of edge points, and G is the feature value assigned to the edge.

**Figure 4:**
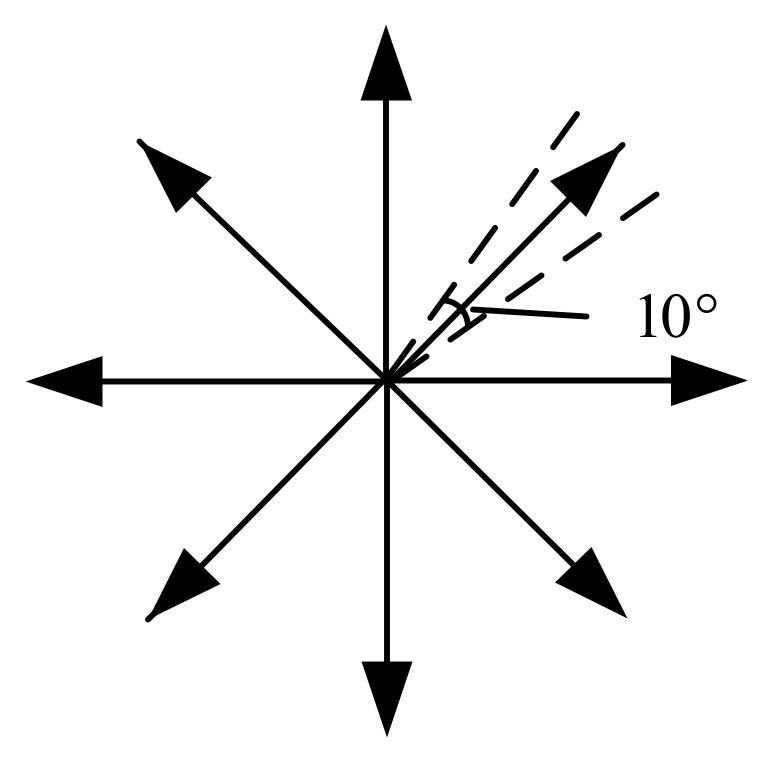
One of the eight regions.

We screen out all the edges that meet the following criteria:

(1) The same edge should be more than six-pixel points to match the basic requirements of ellipse fitting.
(2) The total number of those pixel points should be less than the image pixel circumference.
The pixel points are continuous. To reasonably set up eight regions, we take the pixel center point $\left(\bar{X},\bar{Y}\right)$ and then calculate the angle θ of the edge around the pixel center point and
  (5) $$\theta =\mathrm{fastA}\tan2(X_{k}-\bar{X},Y_{k}-\bar{Y})\times \frac{\pi }{180}$$
  where the function fastAtan2 is used to find the angle. The angle deviation of each region is 10° as shown in Figure 4. If there are edge points in each area, the edge is continuous. The angle setting of the regions cannot be greater than 45°. Otherwise, there will be an intersection between the regions, which will cause the method to fail. Just choose a smaller value, such as 10°.

### 4.3. Edge Fitting

We start elliptic fitting for the edges that meet those conditions described in the previous section. The projection pattern is a parallel projection, where the distance from the Circular-ring marker to the camera’s optical center is much larger than that of the marker size. After parallel projection, the circle appears as an ellipse on the image. In this section, an ordinary least square is used for ellipse fitting [36]. The ellipse equation is expressed as

(6) $$\left\{\begin{array}{l} x^{2}+\lambda_{1}xy+\lambda_{2}y^{2}+\lambda_{3}x+\lambda_{4}y+\lambda_{5}=0\\ \lambda_{1}{}^{2}-4\lambda_{2}<0 \end{array}\right..$$

The five basic parameters of the ellipse are calculated from the ellipse equation as follows

(7) $$\left\{\begin{array}{c} \varphi =\frac{1}{2}\arctan\frac{\lambda_{1}}{1-\lambda_{2}}\\ x_{0}=\frac{\lambda_{1}\lambda_{4}-2\lambda_{2}\lambda_{3}}{4\lambda_{2}-\lambda_{1}{}^{2}}\\ y_{0}=\frac{\lambda_{1}\lambda_{4}-2\lambda_{4}}{4\lambda_{2}-\lambda_{1}{}^{2}}\\ u=\sqrt{\frac{2\left(\lambda_{1}\lambda_{3}\lambda_{4}+4\lambda_{2}\lambda_{5}-\lambda_{2}\lambda_{3}{}^{2}-\lambda_{4}{}^{2}-\lambda_{1}{}^{2}\lambda_{5}\right)}{\left(\lambda_{1}{}^{2}-4\lambda_{2}\right)\left(\lambda_{2}+1-\sqrt{\lambda_{1}{}^{2}+{\left(\lambda_{2}-1\right)}^{2}}\right)}}\\ v=\sqrt{\frac{2\left(\lambda_{1}\lambda_{3}\lambda_{4}+4\lambda_{2}\lambda_{5}-\lambda_{2}\lambda_{3}{}^{2}-\lambda_{4}{}^{2}-\lambda_{1}{}^{2}\lambda_{5}\right)}{\left(\lambda_{1}{}^{2}-4\lambda_{2}\right)\left(\lambda_{2}+1-\sqrt{\lambda_{1}{}^{2}+{\left(\lambda_{2}-1\right)}^{2}}\right)}} \end{array}\right.$$

where $\left(x_{0},y_{0}\right)$ is the pixel coordinate of the ellipse center, u and v are the semi-major and the semi-minor axis of the ellipse. φ is the semi-major axis inclination of the ellipse. For the above-mentioned fitting ellipse algorithm, we can get many ellipses in the image. To exclude some unqualified ellipses, we should ensure that qualified ellipses meet the following three essential conditions.

(1) The ellipse major axis is 1/3 of the image length and width.
(2) The ellipse is inside the image. According to the fitting parameters $x_{0},y_{0},u$, the ellipse is qualified if $\left(x_{0}-u,y_{0}-u\right)$ and $\left(x_{0}+u,y_{0}+u\right)$ are inside the image.
(3) Reject ellipse that are too small. The semi-major and the semi-minor axis length of the ellipse are less than two pixels and thus are considered unqualified. The area of the ellipse should satisfy πuv>12.
  If the ellipse meets all of the above conditions, the next step performs a secondary screening. The specific implementation scheme is to traverse two ellipses at a time. The center pixel coordinates of the two ellipses are $\left(x_{m},y_{m}\right)$ and $\left(x_{n},y_{n}\right)$. The semi-major axis and semi-minor axis are $u_{m},v_{m},u_{n},v_{n}$ and the characteristic values are $G_{m},G_{n}$. We need two ellipses that satisfy the following four constraints.
(4) For the two ellipses, $u_{m}/u_{n}=h$ should hold, where h is the preset threshold, and it depends on the light source intensity. Section 5.1 determines the effective range of the threshold h. The effective range ensures that the correct two ellipses are not deleted by mistake.
(5) The circular ring is composed of a white–black–white region. In cases where the edge is white–black, its characteristic value is greater than zero, and for a black–white edge, its characteristic value is less than zero. For the Circular-ring marker in this article, the feature values $G_{m}$ and $G_{n}$ of the two ellipses should satisfy
  (8) $$G_{m}\times G_{n}<0$$
(6) The outer ellipse size should be more than the inner ellipse size, i.e.,
  (9) $$\left\{\begin{array}{l} (u_{m}-u_{n})\times (G_{m}-G_{n})>0\\ (v_{m}-v_{n})\times (G_{m}-G_{n})>0 \end{array}\right.$$
(7) The pixel distance between the centers of the two ellipses is small enough and
  (10) $$\left\{\begin{array}{l} \left|x_{m}-x_{n}\right|<K\times \max(u_{m},u_{n})\\ \left|y_{m}-y_{n}\right|<K\times \max(u_{m},u_{n}) \end{array}\right.$$
  where *K* is a constant. The constraint range of the central pixel deviation value varies with the size of the ellipse. After verification of all experiments in Section 5, the ratio between the center coordinate difference of the two ellipses and the semi-major axis is less than 0.055. We set *K* to 0.06, and the marker identification effect is strong.

The two ellipses that meet the conditions are recorded with their center pixel coordinates. We take the corresponding average values $\bar{U}=\left(x_{m}+x_{n}\right)/2$ and $\bar{V}=\left(y_{m}+y_{n}\right)/2$. Then, the point $\left(\bar{U},\bar{V}\right)$ is set to the pixel coordinate of the center of the circular ring.

### 4.4. Decoding of Circular Ring

**Figure 5:**
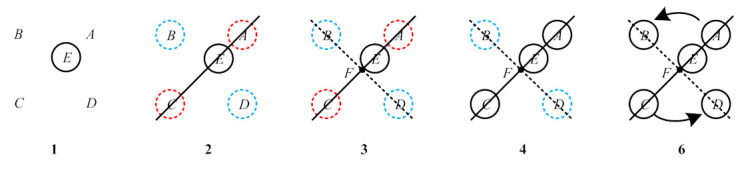
Steps 1, 2, 3, 4, and 6 decode the schematic image.

In the previous section, the Circular-ring marker can be decoded using the center pixel coordinate point of the circular ring that meets the criteria. There are also pseudo-circular rings in the processed image, so the number of center pixel coordinate points we get will be greater than or equal to five. Next, we will assign the exact pixel coordinates for the points A,B,C,D,E in turn:

(1) Find the internal point E from the five points. Then, use the positional relationship between the internal point and the remaining four points so that
  (11) $$S_{A}{}_{BCD}=S_{ABE}+S_{ACE}+S_{ADE}+S_{BCE}$$
  where $S_{A}{}_{BCD}$ is the area of a quadrilateral formed by the four points A,B,C,D, $S_{ABE},S_{ACE},S_{ADE},S_{BCE}$ are the area of the triangle formed by the internal point E and the four sides of a quadrilateral ABCD, respectively. The point that satisfies this equation is an internal point E. Then, point E is assigned pixel coordinates. As shown in No. 1 in Figure 5, point E is represented by a black solid circle.
(2) Find the points A,C that are collinear with the internal point $E({\bar{U}}_{E},{\bar{V}}_{E})$. The two-point construction line is
  (12) px+qy+r=0
  where p,q,r are constants. We calculate the vertical distance L according to Equation (13). The L is the distance from E to line AC.
  (13) $$L=\frac{\left|p{\bar{U}}_{E}+q{\bar{V}}_{E}+r\right|}{\sqrt{p^{2}+q^{2}}}$$
  If L is small enough and $\vec{AE}\cdot \vec{CE}<0$, one can consider the two target points as points A,C. Note that this step finds point A,C, but does not assign pixel coordinates. As shown in No. 2 in Figure 5, this step separates points A,C from points B,D. The circles in different colors in Figure 5 represent different areas, and the dotted circles represent no pixel coordinates assigned.
(3) Determine the pixel coordinate of the Circular-ring marker center point F. By Step 2, we know that the remaining two points are B,D. Then, we find the intersection F between line AC and line BD, as shown in No. 3 in Figure 5. The coordinate of the point F can be obtained by calculation. Point F should satisfy
  (14) $$\left\{\begin{array}{l} \vec{AF}\cdot \vec{CF}<0\\ \vec{BF}\cdot \vec{DF}<0 \end{array}\right.$$
  where $\vec{AF},\vec{CF},\vec{BF},\vec{DF}$ are the directed line segments between two points.
(4) Determine the positions of points A and C. In Step 2, A,C are determined, but they do not assign pixel coordinates, as shown in No. 4 in Figure 5. In the Circular-ring marker, E is between point A and point F, and A satisfies $\vec{EF}\cdot \vec{EA}<0$ and point C satisfies $\vec{EF}\cdot \vec{EC}>0$. Then, under the constraint conditions in this step, points A and C are assigned pixel coordinates values in sequence.
(5) Using the obtained A,E,F,C, we calculate the cross-ratio R(A,E,F,C):
  (15) $$R(A,E,F,C)=\frac{\left|\vec{AF}\right|\times \left|\vec{EC}\right|}{\left|\vec{EF}\right|\times \left|\vec{AC}\right|}$$
  where $\vec{\left|AF\right|},\vec{\left|EC\right|},\vec{\left|EF\right|},\vec{\left|AC\right|}$ are modules of the directed line segments. According to the projective theorem of cross-ratio invariance, R(A,E,F,C)=R(a,e,f,c). Note that image recognition may produce certain errors, so we can set qualifying conditions to specify the allowable error range. If the cross-ratio error ΔR is within the allowable error range, then A,E,F,C satisfy the cross-ratio invariance. Otherwise, the decoding work will go back to Step 1.
(6) Determine the positions of points B and D. In Step 2, B,D are determined, but they do not assign pixel coordinates. We set the point F as the center of the mass and then calculate the value β of the points A,B,C,D relative to the center of the mass:
  (16) $$\left\{\begin{array}{l} \beta =2^{17}-P_{x}/P_{y}\;\begin{array}{c} \begin{array}{c} P_{y}>0,P_{x}<0 \end{array} \end{array}\\ \beta =2^{16}+P_{y}/P_{x}\begin{array}{cc} & \;P_{y}>0,P_{x}>0 \end{array}\\ \beta =-2^{16}+P_{y}/P_{x}\begin{array}{cc} & \;P_{y}<0,P_{x}<0 \end{array}\\ \beta =-P_{x}/P_{y}\begin{array}{cc} & \;P_{y}<0,P_{x}>0 \end{array} \end{array}\right.$$
  where $P_{x}$ is the x-axis difference and $P_{y}$ is the y-axis difference. In Formula (16), the centroid point F can be set as the origin of the coordinate. At this time, the points A,B,C,D should be located in four different quadrants on the coordinate axis. $P_{x}$ is the x-axis difference between the points A,B,C,D and the point F. $P_{y}$ is the y-axis difference between the points A,B,C,D and the point F. Then, calculate the β value of the points A,B,C,D in turn. As shown in No. 6 in Figure 5, in the counterclockwise direction, β is increased, and we can determine the positions of B, D based on the positions of A,C. Then, the points B and D are assigned pixel coordinates values in sequence.

In cases where the decoding Steps 1–6 are not satisfied, we enter the pixel coordinates of the five circular-ring centers in the next group for decoding. If the qualified five circular-ring center pixel points are not found, there is no Circular-ring marker in the image. Otherwise, the decoding is described as successful, and we record A,B,C,D,E and their pixel coordinates.

## 5. Experiment and Analysis

We propose a Circular-ring marker for a single-target mobile robot location. The performance of the proposed location method is evaluated in terms of detection range, light change robustness, and positioning accuracy. We further compare the results with current visual reference markers. We take a 6 DoF manipulator as the experimental mobile platform. The camera model is TS4MCL-180M/C, with the resolution of and a frame rate of 149 FPS. The camera is produced by the i-tek company in Hefei, Anhui, China. The prime lens is HC2505A with the focal length of 25 mm. We experiment with the Circular-ring marker, ArUco, AprilTag, and Checkerboard methods. This Checkerboard uses an adaptive threshold method to find corners. Then, it uses the cornerSubPix function to perform sub-pixel accuracy on the corners.

We use the four corners and the EPnP+LM algorithm to optimize the pose calculation [37,38]. Then, the minimized reprojection error is used to obtain the pose information.

### 5.1. Parameter Setting Experiment

We need to determine the threshold h, which was mentioned in Step 4 in Section 4.3. The theoretical threshold of the circular ring printed on the paper is 2. The threshold h and the camera projection can change. In the experiment, we use a light source of a certain intensity and adjust the exposure time of the camera to control the light source on the photosensitive surface of the camera. We set the exposure time at 700–6400 µs and take ten images every 300 µs. Then, we calculate the average value of the threshold, h for each circular ring.

**Figure 6:**
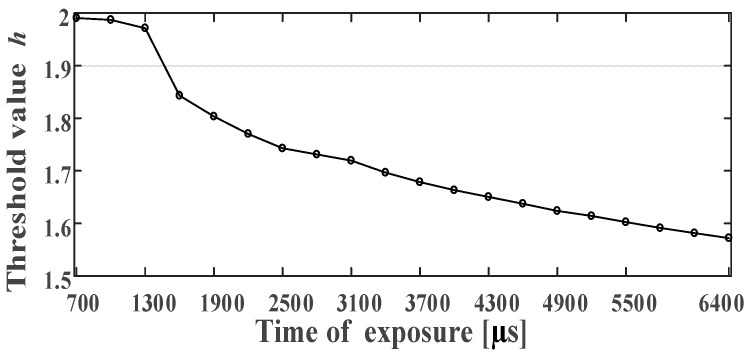
The relationship between the threshold h and the light source intensity.

As it is seen in Figure 6, we can limit the threshold h between 1.55 and 2 to ensure the effective detection of the Circular-ring marker.

In Section 4.3, parameter *K* in Step 7 needs to be determined as well as parameter *L* in Step 2 and parameter ΔR in Step 5 in Section 4.4. They affect the rate of marker identification. In the subsequent identification experiment, the parameter setting considers the experimental values in Table 1. The experimental image is about 6000 frames. Then, we calculate the values of each parameter in the image in turn. Table 1 records the maximum value of each parameter after the marker is correctly identified. As it is seen in Table 1, this maximum value is much smaller than the experimental value, so we can reduce the value of each parameter to prevent misidentification. Finally, we take approximately 110% of this maximum value as the preset value for the follow-up work.

**Figure 7:**
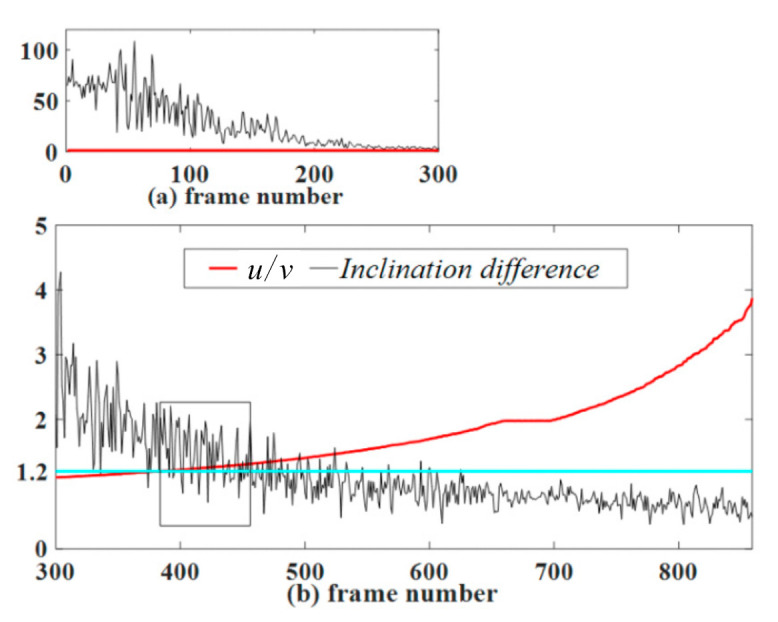
The relationship between this inclination difference Δφ and u/v. The parameter u is the semi-major axis of the outer ellipse. The parameter v is the semi-minor axis of the outer ellipse.

The semi-major axis inclination φ of the ellipse is mentioned in Section 4.3. The Circular-ring marker consists of five circular rings. Theoretically, the inner and outer edges of the circular ring have the same inclination φ. In fact, due to the existence of identification error, the inclinations of the inner edge and the outer edge will have a deviation Δφ. For this reason, we studied the relationship between the inclination difference Δφ and u/v. In the first frame, the marker plane is perpendicular to the camera’s optical axis, and then the marker slowly rotates about 80 degrees along the camera’s Y-axis. The camera captures a total of 859 frames. As shown in Figure 7, the black line represents the inclination difference Δφ. The blue line is the indicator line y = 1.2. As it is seen in Figure 7a, in the first 300 frames, when the u/v value is close to 1, the Δφ value changes too much. After the 300th frame, as u/v increases, the Δφ value tends to stabilize. Therefore, the Circular-ring marker misidentification rate can be reduced by adding restriction conditions. For example, in the subsequent marker identification process, when u/v>1.2, the constraint Δφ<3° must be satisfied.

### 5.2. Effective Distance Detection

We need to verify the impact of distance on the performance of the positioning system. The prime lens used in this experiment can be manually adjusted to generate sharp images. We place the Circular-ring marker 3 m away from the camera and then adjusted the prime lens to make the image sharp. In the subsequent experiments, the prime lens is not manually adjusted again. The size of all markers is 67.5×67.5 mm. In this experiment, the printed multi-markers paper is slowly moved from 0.5 to 7.2 m. The camera is fixed and continuously capture images to obtain a continuous sequence of pictures. In the experiment, the recognition rate is 1 or 0 after each frame is processed. To obtain a smooth data set, we recalculate the position of the robot and consider 30 adjacent frames of the current frame to obtain the average recognition rate.

**Figure 8:**
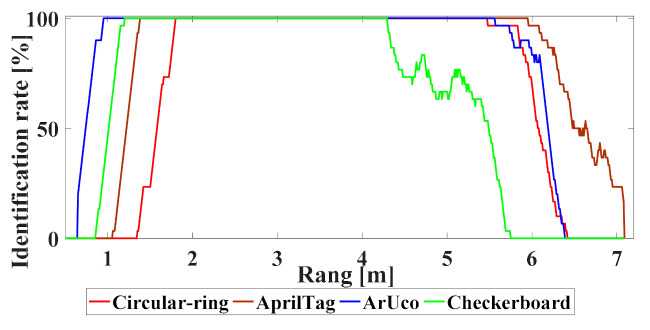
The marker identification rate versus distance.

When each marker is used for robot positioning, as seen in Figure 8 and Table 2, AprilTag stably recognizes within 1.3–6.1 m without misidentification. The ArUco also stably recognized within 0.9–5.8 m, and its detection performance is high at a close range. We are using OpenCV to recognize the Checkerboard, which is stable between 1.1 and 4.3 m; however, it has a short effective range. The new Circular-ring marker can also achieve a stable recognition range of 5.8 m and recognize 1.7 m at a short distance without misrecognition.

### 5.3. Robustness against the Light Changes

**Figure 9:**
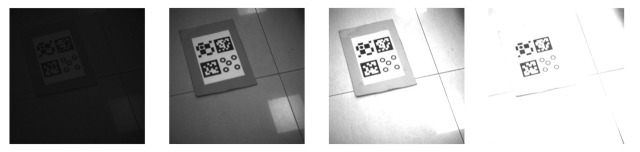
The time of exposure is 100 µs, 1000 µs, 3300 µs, and 6600 µs.

Here, we investigate the recognition effect of markers under different light intensities. To do this, we follow the experimental procedures as described in Section 5.1. We set the exposure time between 100 and 6600 µs to control the light intensity. This method avoids the interference of external natural light with the ambient light. Figure 9 shows the image taken under partial exposure time. Table 3 presents the recognizable time of the exposure range.

In Table 3, AprilTag, ArUco, and Checkerboard demonstrate similar robustness. However, they have poor adaptability and poorly perform under strong light. Although the proposed Circular-ring marker is less adaptable under low light conditions compared to the previous markers, it shows stronger robustness under the strong light conditions.

### 5.4. Detection Speed of the Marker

Here, we determine how quickly the markers can be identified. The sequence images obtained in Section 5.2 are used for time calculations. The C++ library function getTickCount() is used to calculate how long it takes to identify each marker. The results are shown in Table 4.

The marker detection method in each frame is the same. Among these markers, AprilTag and Checkerboard take a longer time. The Circular-ring marker has higher computational efficiency and provides faster processing speed. However, it is slower than ArUco.

### 5.5. Repeatability of the Positioning Experiment

**Figure 10:**
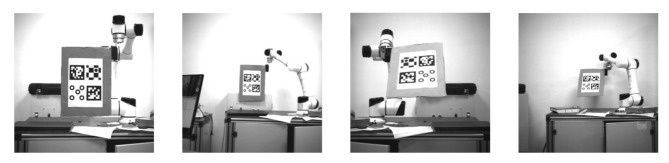
The 0° and 45° angular position images at 2 and 4 m.

The repeatability of the positioning accuracy of the robot was also examined. We used a 6 DoF robotic arm with a rotation accuracy of 0.003° and a translation accuracy of 0.03 mm as a reference. Those markers are fixed at the end of the robotic arm, and the camera is fixed on the platform. The dimensions of these markers are all 90 × 90 mm. We tested the markers at 0° and 45°. The angle was determined using the angle between the vertical direction of the marker and the camera optical axis. The measurement interval was 0.2 m in the range of 2–4 m. Figure 10 shows some experimental pictures. Then, we collected the data at each position for 22 groups and 100 images per group.

**Figure 11:**
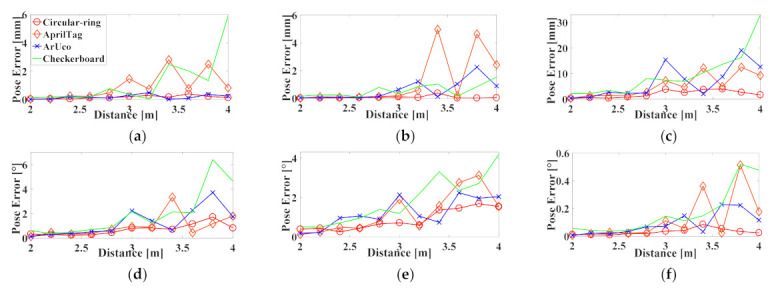
Six DoF correspond to repeatability errors if these markers are at 0°: (**a**) Translation error along X-axis; (**b**) Translation error along Y-axis; (**c**) Translation error along Z-axis; (**d**) Rotation error along X-axis; (**e**) Rotation error along Y-axis; (**f**) Rotation error along Z-axis.

We take the first frame as the initial pose of each group and then obtain the relative poses of other images. The pose is defined as six degrees of freedom $t_{x},t_{y},t_{z}$ and $r_{x},r_{y},r_{z}$. We take the absolute value for each DoF deviation and then calculate their average values. For the cases where these markers are at 0°, the experimental data are shown in Figure 11.

In this paper, when the Circular-ring marker is used for robot positioning, the maximum average errors at each position $t_{x},t_{y},t_{z}$ are 0.398, 0.368, and 3.967 mm, respectively. The $r_{x},r_{y},r_{z}$ maximum average errors are 1.726°, 1.681°, and 0.085°, respectively. Furthermore, the translation error on the *Z*-axis is always greater than the *X*-axis or *Y*-axis. The rotation error on the *Z*-axis is always less than that of the *X*-axis or *Y*-axis. We use Euclidean distance to represent translation error and rotation error. We think that the comparison between Circular-ring marker and other markers is more intuitive. Table 5 records the maximum Euclidean distance of each marker in translation error and rotation error. The calculation formula is as follows:

(17) $$\left\{\begin{array}{c} \Delta t=\sqrt{t_{x}\times t_{x}+t_{y}\times t_{y}+t_{z}\times t_{z}}\\ \Delta r=\sqrt{r_{x}\times r_{x}+r_{y}\times r_{y}+r_{z}\times r_{z}} \end{array}\right..$$

**Figure 12:**
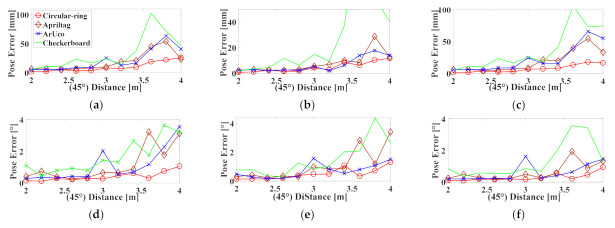
Six DoF correspond to repeatability errors when these markers at 45°: (**a**) Translation error along the X-axis; (**b**) Translation error along the Y-axis; (**c**) Translation error along the Z-axis; (**d**) Rotation error along the X-axis; (**e**) Rotation error along the Y-axis; (**f**) Rotation error along the Z-axis.

The experimental data are presented in Figure 12 where these markers are at 45°. In this paper, when the Circular-ring marker is used for robot positioning, the maximum average errors at each position $t_{x},t_{y},t_{z}$ are 26.053, 11.572, and 18.024 mm, respectively. The maximum average errors of $r_{x},r_{y},r_{z}$ are 1.049°, 1.305°, and 0.930°, respectively. The experimental data suggest that in cases where the Circular-ring marker is at 45°, its average error is worse than the Circular-ring marker at 0°. Finally, the repeatability of the location accuracy of the Circular-ring marker is slightly smaller than that of AprilTag and ArUco. The checkerboard has the worst repeatability of the positioning accuracy.

### 5.6. Positioning Accuracy Experiment

The previous section shows that the repeatability of the positioning accuracy of the Circular-ring marker is very high. The repeatability of the positioning accuracy of the Circular-ring marker was on a par with AprilTag and ArUco, and even our marker effect is better. The Checkerboard have large repeatability errors and shorter recognition distances. The experiment in this section verifies the location accuracy of the Circular-ring marker. At 2 m, the marker moves ±200 mm in steps of 50 mm along both the X and *Y*-axis. At 4 m, the marker moves similarly but it moves 400 mm in steps of 100 mm. At 2 m and 4 m, the marker is rotated 45° along the X-axis, Y-axis, and Z-axis of the camera, respectively. We take pictures every 5°.

**Figure 13:**
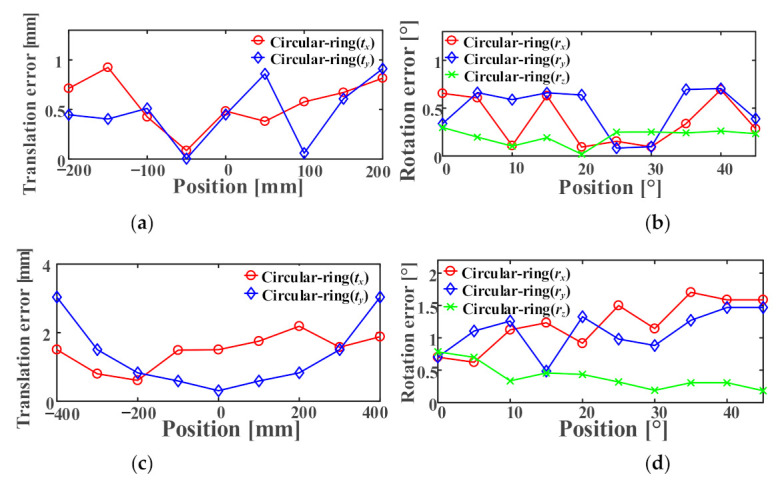
The translation and rotation accuracy of X and Y-axis at 2 m and 4 m: (**a**) Translation error along the X and Y-axis at 2 m; (**b**) Rotation error along the X and Y-axis at 2 m; (**c**) Translation error along the X and Y-axis at 4 m; (**d**) Rotation error along the X and Y-axis at 4 m.

We capture the first frame as the initial pose of each group and then obtain the relative poses for the other images. Using the Euclidean distance to calculate the translation error for each axis, we capture ten images at each position and then calculate the average error. The experimental data are presented in Figure 13.

In Figure 13, the average of the DoF deviation can be solved by its absolute value. When the Circular-ring marker is used for robot positioning at 2 m, the maximum average errors of $t_{x},t_{y}$ are 0.92 and 0.91 mm, respectively, and the maximum average errors of $r_{x},r_{y},r_{z}$ are 0.692°, 0.704°, and 0.296°, respectively. When the Circular-ring marker is used for robot positioning at 4 m, the maximum average errors of $t_{x},t_{y}$ are 2.19 and 3.04 mm, respectively, and the maximum average errors of $r_{x},r_{y},r_{z}$ are 1.703°, 1.468°, and 0.782°, respectively. The results in this section suggest that the farther the camera from the Circular-ring marker, the better to keep the error within the allowable range.

**Figure 14:**
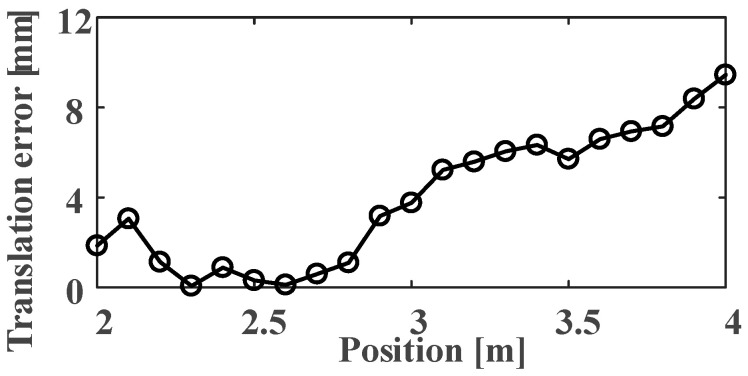
Translation error along the *Z*-axis at 2 to 4 m.

The translation accuracy along the Z-axis is tested. The experimental distance varies from 2 to 4 m, and the movement interval of the Circular-ring marker is 0.1 m. We considered 21 groups and captured ten images per group. In Figure 14, the error $t_{z}$. gradually increases to 9.44 mm by increasing the distance. The experimental data also shows that the Circular-ring marker has a large error at the distance range from 2 to 2.2 m. This might be caused by the small field depth of the used prime lens.

### 5.7. Comparison with Other Visual Positioning Systems

The advantages of the Circular-ring marker for robot positioning are demonstrated by a comparison of its performance with the AprilTag, ArUco, and Checkerboard. In this section, the positioning accuracy under different resolutions, different marker sizes, relative position changes, and greater distances are compared. The angle estimation accuracy is also tested. The final data of each experiment are calculated by the Euclidean distance error described in Section 5.5.

#### 5.7.1. Compare at Different Resolutions

The positioning performance of the four evaluated systems was compared for three different resolutions. All markers are 90 × 90 mm in size. The experiment uses a camera with a resolution of 2048 × 2048. The resolution can be set to 1280 × 1024 and 640 × 480 through the model JHSM130Bs camera. Those markers are located at the same distance in front of the two cameras and attached to the end of the manipulator. Circular-ring, AprilTag, ArUco, and Checkerboard are perpendicular to the optical axis of the camera. Then, the manipulator drives these markers to translate along the *X*-axis of the camera. We record the translation error of each frame in turn.

The results presented in Table 6 indicate that the smaller the image resolution, the lower the positioning accuracy of each marker. When the image resolution is 2048 × 2048, the data show that the positioning accuracy of the Circular-ring marker is about the same as other markers. However, for the Circular-ring marker, the reduction of camera resolution highlights the positioning advantage of the robot.

#### 5.7.2. Comparison of Different Dimensions

**Figure 15:**
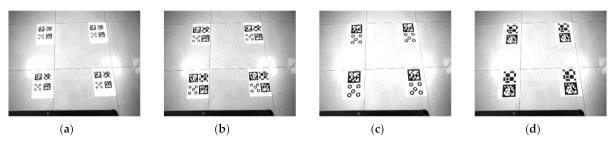
Experiment images under different marker dimensions: (**a**) Markers dimension is 67.5 × 67.5 mm; (**b**) Markers dimension is 90 × 90 mm; (**c**) Markers dimension is 126 × 126 mm; (**d**) Markers dimension is 126 × 126 mm.

The positioning performance of the four evaluated systems was compared for three different sizes. The JHSM130Bs camera was selected in the experiment, and the resolution was set to 1280 × 1024. The markers sizes are 67.5 × 67.5 mm, 90 × 90 mm, and 126 × 126 mm, respectively. In this experiment, the floor tiles form a regular square grid with dimensions of 600 × 600 mm. We use floor tiles as the measurement standard, as shown in Figure 15. After the markers are posted on the ground, the distance between the markers is manually verified, and the error is around 2 mm. The experiment takes 40 frames. We use one of the positions as the initial pose in the image. Then, the relative poses of the other three positions are solved. Finally, we calculate the Euclidean distance error of these four times in turn and then take their average error.

The results presented in Table 7 indicate that the large-sized marker has high positioning accuracy. In the case of the same marker size, the Circular-ring marker is used for robot positioning with higher accuracy.

#### 5.7.3. Comparison of Relative Position Changes

In this experiment, we change the relative position of these markers on the printing paper. Then, we test the positioning accuracy of the four markers. We take 10 frames at each position for a total of 40 frames. The size of the marker used in the experiment is 90 × 90 mm. The subsequent experimental procedures and data processing is the same as in Section 5.7.2.

**Figure 16:**
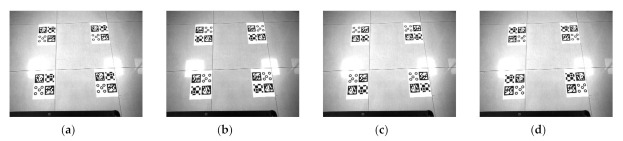
Transform the markers position: (**a**) Position 1; (**b**) Position 2; (**c**) Position 3; (**d**) Position 4.

It can be seen from Table 8 that the positioning accuracy of these markers fluctuates greatly in different positions, especially AprilTag and ArUco. As shown in Figure 16, AprilTag has high positioning accuracy in position 1, and ArUco has high positioning accuracy in position 3. However, as a whole, the positioning accuracy of the Circular-ring marker is still better.

#### 5.7.4. Comparison between Markers at Greater Distances

**Figure 17:**
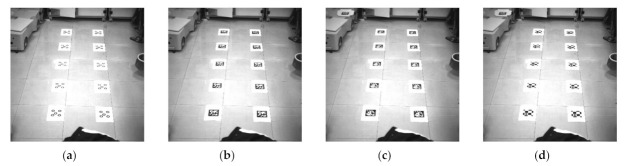
The positioning accuracy experiment of each marker at greater distance: (**a**) Circular ring; (**b**) AprilTag; (**c**) ArUco; (**d**) Checkerboard.

The positioning performance of the four evaluated systems was compared at greater distances. The placement of each marker is shown in Figure 17. We obtained 100 frames in the same experimental environment for a total of 400 frames. We test the positioning accuracy of these markers in the horizontal and vertical directions. In the vertical direction, the marker closer to the camera is used as the initial pose in the image, and then the relative pose of other markers is solved. In the horizontal direction, we use the left marker as the initial pose in the image. Then, we calculate the relative pose of the horizontal right marker.

The results presented in Table 9 indicate that the absolute error of these markers is relatively large, but the relative error data at greater distances show reasonable results. In addition, the positioning accuracy of these markers in the horizontal direction is higher. In the long-distance test experiment, the positioning accuracy of the Circular-ring marker in the horizontal or vertical direction is significantly better than that of AprilTag, ArUco, and Checkerboard.

#### 5.7.5. Accuracy of Angle Estimation

**Figure 18:**
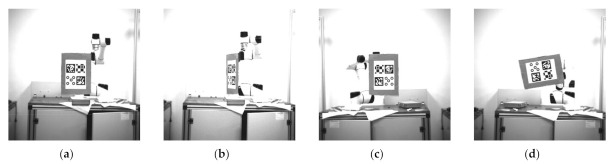
Rotation for angle estimation test: (**a**) The yaw of 0°; (**b**) The yaw of 70°; (**c**) The roll of 0°; (**d**) The roll of 80°.

This section tests the angle estimation accuracy of four evaluation systems. The markers are this time placed at the end of the manipulator, and we use the tilt functions to vary the angle of the markers. As shown in Figure 18, the yaw rotates around the Y-axis of the camera, and the roll angle rotates around the Z-axis of the camera. Then, the record rotations of the markers are compared to the angle taken from the manipulator.

As can be seen from the Table 10, the angle error of the Circular-ring marker is equivalent to that of ArUco, followed by AprilTag and Checkerboard. The Circular-ring marker’s ability to establish the marker’s orientation is successful across all four markers.

### 5.8. Experimental Analysis

In the experiment, the detection rate is improved by setting the effective range of the parameters involved in the process of identifying the marker. For the Circular-ring marker, AprilTag, ArUco and Checkerboard are used for robot positioning. Then, we compare their recognition distance, robustness to light intensity, and time-consuming detection. The results show that the recognition distance of the Circular-ring marker is slightly smaller than that of AprilTag and ArUco. The Circular-ring marker is more robust to strong light, and the detection time is slightly less than that of the ArUco marker. Table 11 records the comparative experimental results among all markers. In the experimental data, the average error of the Circular-ring marker is set as 1, and then the multiple sizes of other markers relative to the average error of the Circular-ring marker are calculated. It can be seen from Table 11 that the average translation error of AprilTag, ArUco, and Checkerboard under different conditions are all greater than twice that of the Circular-ring marker. Only in high-resolution comparison experiment are the average translation errors of all markers approximately equal. Therefore, the Circular-ring marker has a greater advantage in the use of small-resolution images. In addition, the average value of the repeatable rotation error of the Circular-ring marker at a large inclination of 45° is less than 1/2 of the remaining markers. In other cases, the rotation error of the Circular-ring marker is equivalent to that of ArUco, followed by AprilTag and Checkerboard. Finally, the robot is calibrated for translation and rotation accuracy within the range of 2 to 4 m. The result shows that the positioning accuracy of the Circular-ring marker is high.

## 6. Conclusions

We propose a new Circular-ring marker that utilizes the advantage of accurate identification of the center point of the circular ring. In our proposed method, (1) we used the Union-Find algorithm to perform boundary segmentation and then assigned the eigenvalues to all the edges. Then, we used the cross-ratio invariance of the photography theorem to set the unique coding information of the Circular-ring marker. Finally, using experimental analysis, we demonstrated that effective decoding was achieved. (2) We used the least-squares method to fit the ellipse with lower computational complexity. Our analysis confirmed the effect of light intensity on the circular-ring threshold h, which further strengthened the accuracy of the marker recognition. We further investigated the recognition rate and the positioning accuracy of the proposed Circular-ring marker and compared its efficiency with the state-of-the-art visual reference markers. Our experimental results show the effective distance of robot positioning, and our proposed marker is slightly inferior to AprilTag and ArUco but superior to Checkerboard. The Circular-ring marker is equivalent to the state-of-the-art visual reference markers in terms of angle estimation accuracy and robustness to light changes. Furthermore, our experimental results showed in terms of small resolution images, different size markers, and long-distance tests that our proposed robot positioning method significantly outperforms AprilTag, ArUco, and Checkerboard. However, the computational complexity of the proposed Circular-ring marker was slightly higher than that of ArUco. The detection time needed for a Circular-ring marker was 0.077 s, which is enough for achieving real-time performance. In this paper, we also compared the repeatability to measure the robot positioning accuracy of the markers from 2 to 4 m. The results indicated that the proposed Circular-ring marker is twice higher than AprilTag, ArUco, and Checkerboard. Finally, for the distance between 2 and 4 m, we performed translation and rotation experiments to test the accuracy of the robot positioning method in this paper. The maximum average translation error was 2.19, 3.04, and 9.44 mm, respectively. The maximum average rotation error was also 1.703°, 1.468°, and 0.782°, respectively. The experiments suggested that for a single-robot location system, the proposed Circular-ring marker is successful.

The Circular-ring marker in this paper is composed of five rings, which occupies a larger area than other circular markers; that is, the recognition distance is sacrificed under the same size. The five rings of the Circular-ring marker are exactly the same. When the Circular-ring marker is used in multi-robots, the correct acquisition of pose information cannot be guaranteed. In the follow-up work, we try to change the ratio of the inner and outer diameter of each circular ring or set a different cross ratio. It may be possible to obtain the pose information of two to three robots at the same time. In the process of identifying the circular target, it takes a long time to detect the ellipse in the global image. Therefore, we plan to use a faster ellipse detection method, the purpose of which is to improve the real-time performance of the system. The extended research based on the parallel pixel scanning algorithm or the simultaneous multi-direction searching (SMDS) algorithm is one of the ideas for future work.

## Figures and Tables

**Table 1:** The size of each parameter value.

	*K*	*L*	ΔR
Experimental Value	0.2	2 pixels	0.5
Maximum Value	0.055	1.025 pixels	0.297
Preset Value	0.06	1.13 pixels	0.33

**Table 2:** The markers distance range of accurate identification [m].

	Circular-Ring	AprilTag	ArUco	Checkerboard
Identification	1.7–5.8	1.3–6.1	0.9–5.8	1.1–4.3

**Table 3:** The effective range of the exposure time [µs].

	Circular-Ring	AprilTag	ArUco	Checkerboard
Low exposure	400	100	200	100
High exposure	6600	3600	4200	3800

**Table 4:** Average processing time of single marker image [s].

	Circular-Ring	AprilTag	ArUco	Checkerboard
Time	0.077	1.581	0.052	1.347

**Table 5:** The maximum Euclidean distance of translation error and rotation error.

	Circular-Ring	AprilTag	ArUco	Checkerboard
Δt(0°)/mm	3.99	13.56	19.17	33.43
Δr(0°)/°	2.410	3.713	4.205	6.974
Δt(45°)/mm	32.94	81.95	93.23	190.69
Δr(45°)/°	1.915	4.798	4.108	6.651

**Table 6:** Comparison of positioning accuracy under different resolutions.

Relative Error [%]								
	Circular Ring		AprilTag		ArUco		Checkerboard	
	*avg*	*max*	*avg*	*max*	*avg*	*max*	*avg*	*max*
640 × 480	0.43	0.96	1.31	4.29	1.07	4.80	1.35	4.49
1280 × 1024	0.26	0.50	0.88	1.27	0.64	1.84	0.78	1.83
2048 × 2048	0.18	0.32	0.18	0.41	0.39	0.60	0.19	0.39

**Table 7:** Comparison of positioning accuracy under different dimensions.

Mean Error [mm]				
	Circular-Ring	AprilTag	ArUco	Checkerboard
67.5 × 67.5 mm	21.1	41.8	62.0	109.8
90 × 90 mm	6.8	19.5	30.8	32.2
126 × 126 mm	3.4	17.8	7.0	15.3

**Table 8:** Comparison of positioning accuracy under position transformation.

Absolute Error [mm]								
	Circular Ring		AprilTag		ArUco		Checkerboard	
	*avg*	*max*	*avg*	*max*	avg	*max*	*avg*	*max*
1	3.4	8.0	8.8	26.5	29.0	74.4	22.5	62.5
2	2.4	6.1	14.2	46.7	17.4	58.9	23.8	79.0
3	3.8	10.7	19.5	47.2	7.9	17.1	32.2	101.4
4	6.8	17.6	17.0	52.7	30.8	88.2	20.1	58.3

**Table 9:** Comparison of positioning accuracy at greater distances.

		Circular-Ring		AprilTag		ArUco		Checkerboard	
		*avg*	*max*	*avg*	*max*	*avg*	*max*	*avg*	*max*
**vertical**	Absolute error [mm]	101.9	269.4	258.5	436.6	245.0	569.8	284.0	973.0
	Relative error [%]	1.67	4.42	4.24	7.16	4.02	9.34	4.66	15.95
**horizontal**	Absolute error [mm]	48.4	125.9	90.7	243.7	111.8	259.2	134.6	415.4
	Relative error [%]	0.79	2.06	1.49	4.00	1.83	4.25	2.21	6.81

**Table 10:** Errors of angle estimates.

Absolute Error [°]								
	Circular Ring		AprilTag		ArUco		Checkerboard	
	*avg*	*max*	*avg*	*max*	*avg*	max	avg	max
Pitch/Yaw	2.807	4.927	4.011	9.626	3.380	5.157	6.933	9.225
Roll	0.745	1.776	1.948	3.438	0.573	1.490	1.604	3.380

**Table 11:** The ratio of the average error of the different fiducial markers to the Circular-ring marker.

		Circular Ring	AprilTag	ArUco	Checkerboard
**5.5 (translation)**	0°	1	3.4	4.8	8.4
	45°	1	2.5	2.8	5.8
**5.7.1 (translation)**	low	1	3.0	2.5	3.1
	medium	1	3.4	2.5	3.0
	high	1	1	2.2	1.1
**5.7.2 (translation)**	little	1	2.0	2.9	5.2
	medium	1	2.9	4.5	4.7
	big	1	5.2	2.1	4.5
**5.7.4 (translation)**	horizontal	1	1.9	2.3	2.8
	vertical	1	2.5	2.4	2.8
**5.5 (rotation)**	0°	1	1.5	1.7	2.9
	45°	1	2.5	2.1	3.5
**5.7.5 (rotation)**	Pitch/Yaw	1	1.4	1.2	2.5
	Roll	1	2.6	0.8	2.2

## Data Availability

The data used to support the findings of this study are available from the first author upon request.
